# Environmental supportiveness for physical activity in English schoolchildren: a study using Global Positioning Systems

**DOI:** 10.1186/1479-5868-6-42

**Published:** 2009-07-17

**Authors:** Andrew P Jones, Emma G Coombes, Simon J Griffin, Esther MF van Sluijs

**Affiliations:** 1 School of Environmental Sciences, University of East Anglia, Norwich, NR4 7JT, UK; 2 Medical Research Council Epidemiology Unit, Institute of Metabolic Science, Box 285, Addenbrookes Hospital, Hills Road, Cambridge, CB2 0QQ, UK

## Abstract

**Background:**

There is increasing evidence that the environment plays a role in influencing physical activity in children and adults. As children have less autonomy in their behavioural choices, neighbourhood environment supportiveness may be an important determinant of their ability to be active. Yet we know rather little about the types of environment that children use for bouts of physical activity. This study uses accelerometery and global positioning system technologies to identify the charactieristics of environments being used for bouts of continuous moderate to vigorous physical activity (MVPA) in a sample of English schoolchildren.

**Methods:**

The study used a convenience sample of 100 children from SPEEDY (Sport, Physical activity and Eating behaviour: Environmental Determinants in Young people), a cohort of 2064 9–10 year-olds from Norfolk, England, recruited in 2007. Children wore an ActiGraph GT1M accelerometer and a Garmin Forerunner 205 GPS unit over four consecutive days. Accelerometery data points were matched to GPS locations and bouts (5 minutes or more) of MVPA were identified. Bout locations were overlaid with a detailed landcover dataset developed in a GIS to identify the types of environment supporting MVPA. Findings are presented using descriptive statistics.

**Results:**

Boys were also more active than girls, spending an average of 20 (SD 23) versus 11 (SD 15) minutes per day in MVPA bouts. Children who spent more time outside the home were more active (p = 0.002), especially girls and children living in rural locations (both p < 0.05). Children tended to be active close to home, with 63% of all bout time occurring inside neighbourhoods, although boys (p = 0.05) and rural children (p = 0.01) were more likely to roam outside their neighbourhood. Amongst urban children, gardens (28% of bout time) and the street environment (20%) were the most commonly used environments for MVPA bouts. Amongst rural children farmland (22%) and grassland (18%) were most frequently used.

**Conclusion:**

The study has developed a new methodology for the identification of environments in which bouts of continuous physical activity are undertaken. The results highlight the importance of the provision of urban gardens and greenspaces, and the maintenance of safe street environments as places for children to be active.

## Background

Physical activity is associated with a wide range of health benefits [1] and habits developed in childhood are likely to carry through to later life [2]. Physical activity levels in children are deemed to be insufficient [3,4] and are known to decline with age [5,6]. Physical activity promotion in children is therefore currently one of the key strategies of many Western governments, and has been marked by several policy publications, such as 'Healthy Weight, Healthy Lives' [7]. However, there is a lack of evidence of effect of previous promotion efforts [8] and a more profound knowledge of why large numbers of children are insufficiently active is needed.

There is increasing evidence that the environment plays a role in influencing physical activity in children and adults [9]. As children have less autonomy in their behavioural choices than adolescents and adults, the supportiveness of their local environment may be a particularly important determinant of their ability to be active [10]. There is preliminary evidence that those who are allowed outside unaccompanied behave in a more autonomous and exploratory manner and are more active [11,12]. Furthermore, unstructured play has been shown to be an important contributor to overall physical activity levels in children [13]. Yet childhood independence is declining, with heightened concerns over safety resulting in children having less freedom to leave the home unaccompanied [14], and potentially restricting their ability for free play in the neighbourhood.

Key challenges to increasing our understanding of the influence of the environment on children's physical activity have been the difficulties of quantifying how children make use of their local surroundings and the limitations of measures of activity. The recent development of lightweight and precise Global Positioning Systems (GPS) and the use of accelerometers provide a solution as they allow children's locations and intensity of activity to be monitored continuously for longer periods, in a variety of environments and with little intrusion to their normal routines. GPS have recently been used to examine the routes children take to school [15] and patterns of active travel [16,17]. Yet few studies have combined GPS, accelerometers, and a detailed environmental database to assess associations between children's activity patterns and environmental characteristics. Our aim was to identify patterns of use of neighbourhood environments for objectively-measured physical activity in children and thus to identify land uses that appear supportive for children's activity.

## Methods

### The study population

The study used a convenience sample recruited from the existing SPEEDY (Sport, Physical activity and Eating behaviour: Environmental Determinants in Young people) cohort. SPEEDY is a study of environmental, socio-cultural, biological, and psychological influences on children's activity levels and dietary behaviour in a population-based sample of 2064 9–10 year-olds from Norfolk, United Kingdom. Details of the cohort are provided elsewhere [4].

Participants from 14 of the 92 participating schools were invited to take part. Schools were selected in order to create environmental heterogeneity. Per school, all children participating in the original SPEEDY study were invited by post. Parents were asked to return a signed consent form also indicating their holiday plans (as data collection was conducted outside of term time between July and October 2007). Recruitment continued until the target sample of 100 children was reached. In total, 368 children were invited, 224 (61%) did not respond, 32 (9%) declined, and 12 (3%) were not able to participate due to other reasons.

### Data collection

A researcher visited each child and their parents at home to explain study procedures and hand out monitors. Children were handed the previously validated hip worn ActiGraph GT1M accelerometer [18], set at a 5-second epoch. The accelerometer recorded continuously and as the estimation of physical activity prevalence was not the purpose of the study, children were not required to wear the unit for a minimum amount of time each day in order to provide valid data.

Children also received a wrist worn Garmin Forerunner 205 GPS unit, set at an adaptive setting whereby latitude and longitude locations were recorded when the child changed direction or speed. This resulted in a point being recorded every 1 to 10 seconds with a spatial accuracy of approximately 3 meters. Based on recommendations of Trost *et al*. [19], children were instructed to wear both monitors during waking hours on four consecutive days including the weekend. As the battery life of the units was limited, children were asked to not switch the GPS on until they first left their home, and to switch it off when they returned home at the end of each day. The units remained powered on between these times. Children and parents were informed that the battery of the GPS required recharging overnight. Children were also asked to complete an activity diary recording times and reasons when they needed to remove the monitors.

### Data processing

**Figure 1:**
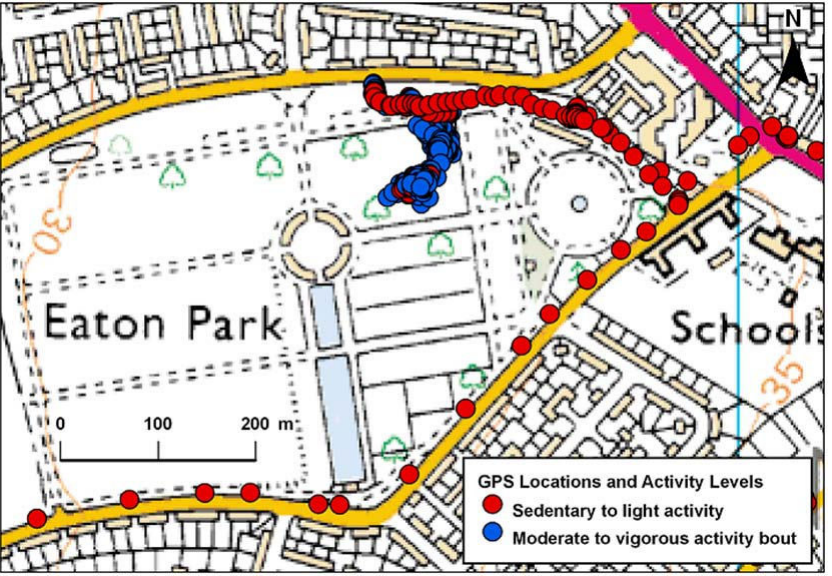
**An example of a child visiting an urban park to play**. Data Crown Copyright ^© ^Ordnance Survey. Used under license.

**Figure 2:**
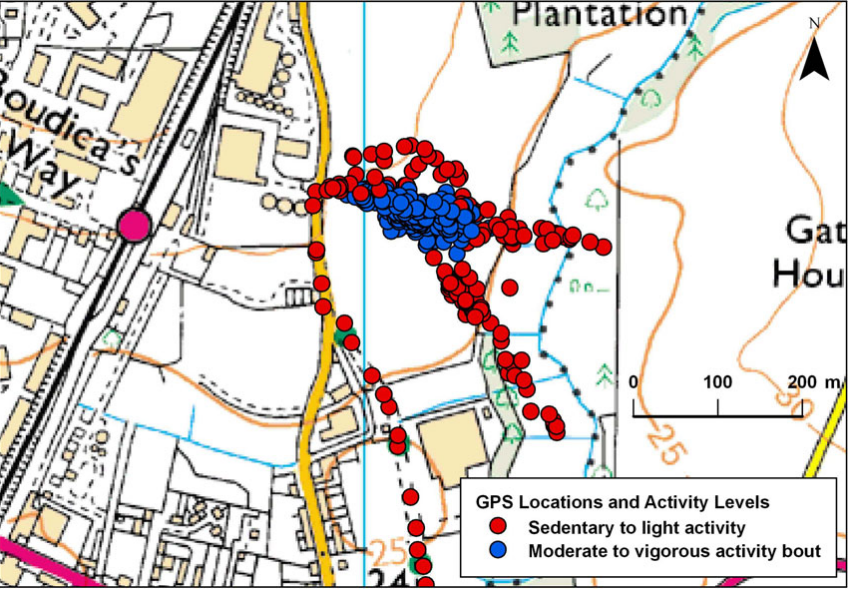
**A child undertaking unstructured play and exploration of uncultivated land**. Data Crown Copyright ^© ^Ordnance Survey. Used under license.

Software was written in Java to match accelerometery data points to the closest recorded GPS location based on their date and time-stamps. Matching was only made if the time difference between the two sets of points was <30 seconds. Periods longer than this were coded as missing because the child might have moved to a new unrecorded location. Each accelerometery data point was then classified into one of 4 intensity categories: sedentary (equivalent to ≤ 100 counts per minute (CPM)), light (101–1999 CPM), moderate (2000–3999 CPM), or vigorous activity (≥ 4000 CPM) [20]. From this, bouts of activity of different intensity were identified. A bout was defined as a period in which a child engaged in physical activity of a given intensity for 5 minutes, allowing up to 1.5 minutes (or 30% of bout time) to be below this threshold [21]. We identified bouts rather than the entire time the children spent above the threshold activity level, as we wanted to identify those environments that were particularly supportive of longer periods of activity, which may be particularly beneficial to health [22]. For this analysis, only bouts of moderate to vigorous physical activity (MVPA) were studied. Figures 1 and 2 show mapped examples of the output produced, with children playing in an urban park and uncultivated land on the periphery of a town.

### Environmental characterisation

The processed data points were entered into the ArcGIS 9.2 Geographic Information System (GIS). This contained a map of land use in the study area derived from Ordnance Survey MasterMap and Centre for Ecology and Hydrology (CEH) Land Cover Map of Great Britain datasets. This provided computer maps of a variety of land uses including building locations, areas of other built land, roads and pavements, private gardens, parks, farmland, grassland, woodland, and beaches. Buildings included domestic residences, shops, indoor sports facilities, and any other covered structures, whilst the 'other built land' category included features such as car parks and yards, hard surface play areas, and pedestrianized thoroughfares.

The neighbourhood around the residential home location of each child was also delineated in the GIS using their exact address. Home locations were identified using the Ordnance Survey Address Point database, and the surrounding neighbourhood was defined as the area within 800 metres along the pedestrian network (roads plus designated public footpaths) of this point. This distance equates to an approximate 10 minute walk.

#### Analytical methods

A series of spatial queries were undertaken in the GIS to identify data points that were part of a bout of MVPA, and extract details of the corresponding land use based on the land use type of the land parcel within which the GPS location fell. The locations were also overlaid with neighbourhood boundaries to identify those falling inside and outside the neighbourhood. Where missing data were present, activity diaries were consulted to determine if the GPS or accelerometer had been removed or if the GPS was unable to determine location due to the presence of buildings or vegetation blocking the satellite signal.

Times when participants were outside their home were identified from the periods during which valid GPS data were available (i.e. the unit was switched on) and showed the children were not in a building. Participants were divided into two categories according to whether they spent an above or below average percentage of their time (found to be 30%) outside of their home compared to the other children.

#### Statistical analyses

Descriptive data were summarised as mean values with standard deviations or percentages. Differences between activity levels observed during the main SPEEDY study and this subsample were tested using independent samples t-tests. Previous studies have shown that the locations in which children are active are modified by child gender [15] and urban/rural status [14], and therefore we also stratified our results according to sex and home location. Differences between boys and girls, and urban/rural status, were examined using Pearson Chi-squared tests. We also examined the interactions between child gender and urban/rural status.

## Results

### Study population

In total 100 children participated; 47 male and 53 female. Overall the sample were not significantly different from the main SPEEDY cohort in terms of their Socioeconomic Status (p = 0.30), urban/rural status (p = 0.29), BMI (p = 0.10), or minutes spent in MVPA (p = 0.49). During the current data collection, the subsample accumulated 12 minutes of MVPA less per day than during the main SPEEDY study (mean = 62 mins, SD = 23 mins, p = 0.004). Table 1 provides a description of the characteristics of the subsample.

**Table 1:** Descriptive personal, anthropometry, and physical activity data on the sample

	**Boys **(n = 47)	**Girls **(n = 53)	**Total **(n = 100)
**SES: parental education (%)**			
- GCSE or lower	34.7	24.5	29.5
- Up to A level	43.7	32.7	37.9
- Higher education	21.6	42.8	32.6
			
**Home location (%)**			
- Urban (>10K)	46.8	47.2	47.0
- Town and fringe	21.3	20.8	21.0
- Village	29.8	22.6	26.0
- Hamlet and isolated dwelling	2.1	9.4	6.0
			
**BMI (kg/m^**2**^) mean (SD)**	17.4 (2.2)	18.0 (3.1)	17.7 (2.7)
			
**IOTF weight status category (%)**			
- Underweight	0.0	0.0	0.0
- Healthy weight	83.0	75.5	79.0
- Overweight	17.0	20.7	19.0
- Obese	0.0	3.8	2.0
			
**MVPA (CPM) mean (SD)**	649.0 (215.4)	595.9 (282.9)	621.3 (252.9)
			
**MVPA (mins) mean (SD)**	70.6 (27.0)	54.8 (16.0)	62.4 (23.3)
			
**Bout time (mins) mean (SD)**	19.8 (22.8)	10.9 (15.1)	15.1 (19.6)

SES = Socioeconomic status; BMI = Body Mass Index; IOTF = International Obesity Task Force; MVPA = Moderate to vigorous physical activity

The children wore the monitors for similar lengths of time (p = 0.112), with boys wearing them for an average of 11.1 (SD 2.2) hours per day, and girls 10.0 (SD 3.2) hours. Overall, locational data from the GPS were available for 66% of all bouts. The GPS were operational but unable to receive a satellite signal in just 0.3% of bouts (which were excluded from analysis) and were presumed to be switched off for the remaining 33.7%. The activity diaries showed this was often because the children were participating in organised sports and were requested to remove the monitors for safety reasons by their instructors.

### Environmental characteristics of physical activity locations

Table 2 shows the average number of minutes per child spent in a bout of MVPA, stratified by percentage of their time spent outside their home. Children who spent more time outside the home were more active (p = 0.002), with this difference being particularly large for girls and children living in rural locations (both p < 0.05).

**Table 2:** The mean length of time (mins) per child (across the four study days) spent in bouts of MVPA according to the time spent outside the home, with standard deviations given in brackets.

	**Gender**		**Home Location**		**Total** (n = 100)
		
	Boys (n = 47)	Girls (n = 53)	Urban (n = 68)	Rural (n = 32)	
**Time outside the home**					
Children spending above average time outside	57.1 (39.9)	28.8 (31.3)	39.1 (29.2)	58.0 (53.1)	45.1 (38.8)
Children spending below average time outside	47.6 (51.1)*	17.0 (24.4)*	32.8 (43.0)*	17.1 (24.1)*	27.7 (38.3)*

* Statistically significant difference between activity levels at p < 0.05.

Table 3 shows the minutes (and percentage) of MVPA bout time spent inside or outside neighbourhoods and in different land use types. Children tended to be active close to home, with 62.5% of all activity bout time occurring inside neighbourhoods. The mean length of time spent in activity bouts inside and outside participants' neighbourhoods was significantly different for boys and girls (p = 0.05), and urban and rural children (p = 0.01) with boys and rural children engaging in higher proportions of MVPA bouts outside the neighbourhood. There was a statistically significant interaction between gender and home location such that urban boys were more likely than gir ls to undertake bouts out of their neighbourhood (p < 0.01). Table 3 also shows that gardens and the street environment supported the greatest amount of bout time, and this was especially so amongst urban children. Both sex (p < 0.001) and home location (p < 0.001) were found to moderate the effects of land use. The fitting of interactions showed that in rural areas, boys made particular use of farm and grasslands, whilst girls were more active in built environments (p < 0.001).

**Table 3:** Mean length of time (mins) per child (across the four study days) spent in bouts of MVPA and standard deviation, according to location relative to neighbourhood boundaries and land use type.

	**Gender**		**Home Location**		**Total** (n = 100)
		
	Boys (n = 47)	Girls (n = 53)	Urban (n = 68)	Rural (n = 32)	
Neighbourhoods*					
Inside neighbourhood	34.9, SD 34.7 (60.4%)	16.0, SD 22.3 (67.0%)	25.7, SD 27.1 (65.0%)	23.1, SD 35.4 (57.2%)	24.9, SD 30.1 (62.5%)
Outside neighbourhood	22.9, SD 33.3 (39.6%)	7.9, SD 13.0 (33.0%)	13.8, SD 26.8 (35.0%)	17.3, SD 22.8 (42.8%)	14.9, SD 25.7 (37.5%)
Land use**					
Buildings	4.1, SD 6.9 (7.0%)	1.7, SD 8.1 (6.9%)	3.4, SD 7.0 (8.6%)	1.5, SD 2.9 (3.7%)	2.8, SD 6.0 (7.0%)
Other built land use	6.5, SD 11.5 (11.3%)	4.6, SD 10.3 (19.4%)	6.5, SD 11.6 (16.4%)	3.5, SD 8.1 (8.6%)	5.5, SD 10.7 (13.9%)
Roads and pavements	10.4, SD 14.5 (18.0%)	4.9, SD 8.2 (20.6%)	7.9, SD 11.0 (20.0%)	6.7, SD 12.9 (16.5%)	7.5, SD 11.7 (18.9%)
Gardens	14.6, SD 21.0 (25.4%)	5.1, SD 9.5 (21.4%)	11.0, SD 15.5 (27.6%)	6.6, SD 17.6 (16.4%)	9.6, SD 16.5 (24.0%)
Parks	3.8, SD 11.2 (6.6%)	2.1, SD 9.3 (8.8%)	2.3, SD 8.9 (5.8%)	4.2, SD 11.6 (10.4%)	2.9, SD 10.0 (7.3%)
Farmland	9.3, SD 19.9 (16.0%)	2.0, SD 6.8 (8.2%)	3.8, SD 12.4 (9.6%)	8.8, SD 18.1 (21.9%)	5.4, SD 14.8 (13.6%)
Grassland	7.5, SD 17.4 (12.9%)	2.3, SD 5.2 (9.5%)	3.6, SD 5.7 (9.0%)	7.1, SD 20.0 (17.7%)	4.7, SD 12.7 (11.8%)
Woodland	1.5, SD 2.9 (2.6%)	0.9, SD 2.9 (3.9%)	1.1, SD 2.9 (2.8%)	1.4, SD 2.6 (3.5%)	1.2, SD 2.8 (3.0%)
Beaches	0.1, SD 0.8 (0.2%)	0.3, SD 2.7 (1.3%)	0.1, SD 0.6 (0.2%)	0.5, SD 2.8 (1.3%)	0.2, SD 1.7 (0.5%)

Column percentages are given in brackets.

SD = Standard deviation. Statistically significant difference between boys and girls, and urban and rural children at *p < 0.05 and ** p < 0.001

## Discussion

In undertaking this study we aimed to provide new evidence on the nature of the environments in which children are physically active. Despite growing evidence from cross sectional studies suggesting that certain environmental characteristics are associated with children's physical activity levels, there is little data characterising the types of place that children actually use to be active.

Our findings show that children who spent more of their time outside their home were more physically active, with the effect being particularly large for children living in a rural environment and for girls. A number of other recent studies have found that children who spend more time outdoors are more active [23,24], this study adds to that by showing that time spent outside the home in general is more actively spent. The association with rurality we observed may be linked to a more diverse range of opportunities for informal play afforded by the rural environment. Lower levels of activity were observed in girls than boys, and this may be because they spend less time than boys outside the home and are especially likely to engage in sedentary activities when indoors.

We found that much activity occurs within an approximate 10 minute walk from home and, perhaps unsurprisingly, this local environment was particularly significant for urban dwellers. An obvious explanation is that the characteristics of the urban environment act to restrain the freedom of children's movement. It may also, in part, be due to differences in the shape and spatial extent of the neighbourhoods we defined, which were based on 800 m distances along the pedestrian network; lower network connectivity in rural areas did lead to generally more linear neighbourhood boundaries, and rural children may hence have been more likely to use informal tracks or paths in order to venture beyond their neighbourhood boundaries.

The Government action plan Choosing Activity [25] and the recently published NICE public health guidance 17 [26], stress the importance of active free play in well-maintained open spaces, and our findings reinforce this. Children were found to use a variety of environments as activity locations, but gardens and street environments appear especially important, together accounting for over 40% of all bouts of moderate to vigorous physical activity in our sample. The particularly high use of these places by urban children may reflect poorer access to the countryside, and may also be a consequence of urban children's more restricted freedom compared to their rural counterparts resulting from heightened safety concerns amongst parents [14].

The apparent importance of gardens for physical activity amongst children has significant policy implications. As demand for housing has risen, the average size of gardens in the UK has reduced. Furthermore, new housing developments have often not provided the same level of community green space as that found within older developments [27]. Our findings suggest these trends may be deleterious if they result in reduced provision of opportunities for children to be physically active. It is of concern that whilst UK housing policies, such as Planning Policy Statement 3 [28] now stipulate that community green spaces and private gardens should be built into new developments, no minimum requirements are specified to ensure that these guidelines are adequately met. In addition, there is presently no formal framework to support the incorporation of recreational spaces into existing build which currently has poor provision [29].

Although the street environment is commonly not thought of as being supportive of physical activity, our findings illustrate that it is an important space that children use to be active, as almost one fifth of bout time took place on roads and pavements. This highlights the need to provide safe residential streets that are suitable for use by children to undertake informal physical activity. The UK Government's *Manual for Streets *[30] has recently outlined plans for changes in urban street design, with an emphasis on the modification of residential roads to primarily support use by pedestrians. Our results suggest there might also be wider physical activity benefits.

This study has a number of strengths, including collecting data during the summer when children have freer schedules and therefore more potential freedom to use the environment in an unrestricted manner, recruiting a sample that was representative of Norfolk children and heterogeneous in environmental exposure, the use of objective measures to assess both dependent and independent variables, and linking of the physical activity and locational data. Notable limitations are the small sample size and missing location data for 34% of the activity bouts. As the activity diaries showed the GPS devices were often removed when participating in team sports and whilst swimming, it is likely that the types of environment in which these activities take place, such as parks and sports centres, are underrepresented. Furthermore, accelerometers are known to poorly measure activity levels during cycling [31] and hence the environments that support cycling may have been underrepresented. The fact we only surveyed during the summer also means we are unable to comment on the supportiveness of the environment during colder and wetter months when children spend less time outside, and may be less likely to use soft, potentially waterlogged, surfaces [32].

There are a number of further limitations. Logistical considerations limited our sample size to 100 children which restricted our power to test multiple hypotheses in the study. As one of the aims of the work was to detect key environments which were supportive of physical activity in the children, we defined a set of bout criteria which only identified periods of MVPA which lasted for 5 minutes or more. A consequence is that our analysis excludes very short periods of MVPA, and hence our findings do not consider the entire period the children spent meeting our activity intensity threshold. Although the SPEEDY cohort was recruited to maximise environmental heterogeneity, Norfolk does not contain any large urban conurbations and has relatively few areas of high deprivation.

Further work in more heterogeneous locations and amongst different ages groups will provide insight into whether the activity patterns observed here are representative of children's behaviours more generally. In addition, an extension to this work will be to link the output from this type of analysis to the types of environmental measures that have commonly been examined in cross sectional studies, such as road densities, access to facilities, and indicators of social capital, to evaluate if these do appear to mediate children's physical activity patterns.

## Conclusion

We found that children make considerable use of informal environments such as urban streets and rural grasslands as locations in which to undertake bouts of moderate to vigorous physical activity. Our findings thus illustrate the importance of the area around the home for children's physical activity. Although children often engage in informal play, and do not necessarily always make conscious decisions to be active, it is vital that safe and supportive environments are available within communities to facilitate this and help them develop good physical activity habitats. It is therefore important to develop appropriately designed and implemented policies that provide the types of environment that will promote informal physical activity in children.

## Competing interests

The authors declare that they have no competing interests.

## Authors' contributions

AJ Conceived of the study, and participated in its design and coordination and helped to draft the manuscript. EC participated in the study design, undertook data collection and analysis, and helped to draft the manuscript. EVS participated in the study design, assisted in its coordination, and helped to draft the manuscript. SG participated in the study design and critically revised the manuscript.
